# Cumulative Health Drivers of Overnight Hospitalization for Australian Working-Age Adults Living Alone: The Early Warning Potential of Functionality

**DOI:** 10.3390/ijerph192214707

**Published:** 2022-11-09

**Authors:** John Rodwell

**Affiliations:** Department of Management & Marketing, Swinburne University of Technology, Hawthorn, VIC 3122, Australia; jrodwell@swin.edu.au

**Keywords:** hospitalization, living alone, accumulation, health trajectory, physical functioning, health insurance, work ability

## Abstract

There is a need to better understand the drivers of hospital utilization for the large and growing number of adults living alone. The cumulative effect of health drivers can be assessed by initially considering clinically advised information, then considering issues that a general practitioner or the person themselves may know. Logistic regression analyses were conducted on longitudinal data from the Household, Income, and Labor Dynamics in Australia (HILDA) survey with three time points over four years (n = 1019). The significant predictors of overnight hospitalization were the presence of a long-term health condition (Time 1), hospitalization severity and comorbidity (Time 1), work ability (Time 2), physical functioning (Time 2), being separated/divorced and having one or more health care cards. Health issues were predictive up to four years before the hospitalization window. That baseline risk of hospitalization was modified as symptoms and relatively salient changes in functionality accumulated. Specific sub-groups of hospital users had access due to insurance or special coverage. The impact of living alone on hospitalization may be able to be partly addressed through interventions such as improving access to primary care and using early warning triggers such as decreasing functionality to seek primary care before seeking hospitalization.

## 1. Introduction

The number of one-person households in Australia has almost doubled over the 25 years from 1991 to 2016 [1] and is projected to increase in number by another 60 percent between 2016 and 2041 [2]. The increasing number of lone-person households means that there is less informal care available within households, and those who live alone may rely more on institutional health services, particularly hospitalization [3]. Hospitalization is a focus here because in-patient care is often the largest portion of health services expenditure [4], with Australia’s health expenditure in 2019–2020 distributed across health services with 41.2% on hospitals, 33.1% primary care, and 10% on referred medical services, with the remaining 15.3% on other services, research and capital spending [5].

There is limited, conflicting evidence as to whether living alone impacts the utilization of health services [6]. Some studies have found links between living alone and hospitalization (e.g., [3]), while others have not (such as [4]), and many of the studies that do look at those issues focused on people over 65, or over 70, years of age (e.g., [7]). Yet with a wide array of health care support available from 65 years of age and, since 2000 in Australia, potential penalties for those aged 30 years of age and older without private health insurance (for more details, see [8]), an under-studied cohort is those living alone aged 30 to 64 years. A similar delineation of adulthood, with a focus on those aged 30 to 64 years, has been used in related studies (such as [9]). This age range may also reflect the ‘second age’—the age of independence, maturity, responsibility and earning, occurring after the first age of socialization and education [10]. Consequently, there is a need for a more detailed examination of the drivers of hospitalization for those who live alone, especially among more of a post-education adulthood or second-age population.

Individuals who live alone are considered to be socially isolated, and although social isolation is related to loneliness, the two are distinct [11]. Social isolation is more of an objective state, whereas loneliness is more of a qualitative, subjective assessment [12]. By focusing on living alone as a proxy for social isolation [11], studies can investigate the possible mechanisms by which living alone may impact hospitalization. The two main mechanisms for the impact of living alone appear to be either through social isolation resulting in poor health and increased health needs or simply that those living alone may require increased support, sought through hospitalization, during episodes of ill health [6].

Studies of the impacts of living alone tend to focus on a specific condition or issue, such as falls [13], usually among the elderly. However, developing trajectories for any given disease within the group of adults living alone may lead to the number of cases being too small to be statistically stable and a large number of trajectories. Other studies have suggested that it may be worthwhile to categorize the nature of prior health conditions. For example, the impact of prior health issues has been considered in terms of hospitalization due to conditions categorized by the extent to which they could be prevented by primary care interventions [11] or classed as planned relative to unplanned hospital admissions [14], although, again, their samples were older people.

A more inductive approach would generate group-specific drivers of hospitalization. In terms of predicting hospitalization for those living alone, common serious illnesses could be categorized in terms of hospitalization likelihood and whether or not the person has comorbidity among the serious illnesses while directly considering how that sub-group of the population may react, whether clinically appropriate or not. Further, sub-groups of the population, such as those living alone, may not follow guidelines to go to primary care when in a potentially distressed state during a health event, instead going to a hospital (applying [6]). Thus, such inductively generated classifications, in terms of drivers of hospitalization for those living alone, may also be indications of dysfunctions in the healthcare system [14].

Categories of health indicators can show the state of the person’s health some years prior to the target period. The health status may be built upon in terms of further, accumulated health assessments in order to be able to delineate health trajectories and assess the cumulative effects on health, possibly reflecting clinical trajectories, usually assessed for a specific disease profile (e.g., sepsis [15]), but in this case at a broad level.

Building a cumulative health profile of those living alone entails starting with health indicators from some time before the hospitalization window. That separation in time between the drivers and the target window may also clarify the nature of the trajectory to hospitalization for those who live alone. In contrast, previous studies of those living alone have often used health variables from the same period as the hospitalization, which may be biased and represent reverse causation because the measure of health state may have occurred after the hospitalization event [3]. Therefore, in order to separate likely health drivers from the overnight hospitalization event, previous health issues can be considered some time before the hospitalization. The accumulating health issues could come from a variety of indicators ranging from the more general to the more work-oriented and in terms of whether certain conditions are associated with hospital stays, even if the hospital stay could have been prevented by primary care treatment (per [11]).

Consequently, this study will investigate the impact of key drivers of overnight hospitalization for those who live alone, considering an array of health details. Such a cumulative health profile could represent issues to consider for those living alone prior to considering mortality, the focus of other studies (e.g., [16]).

To begin building a health trajectory for adults living alone, this study considers previous serious and long-term health conditions that the person is likely to know from four years prior to the period examined for overnight hospitalization. Then, a year prior to the target window, more general, overall facets of health can also be considered in terms of physical functioning, general health and mental health. Such indicators of health-related quality of life, particularly using tools such as the SF36 [17], have often been used to inform the assessment of health trajectories [18]. Similarly, functional impairment has been found to predict hospitalization and mortality for people over 65 years of age [19], with self-reported physical functionality predicting hospitalization for the elderly [20].

Closer to the event window being analyzed, people are likely to be able to assess the extent to which their health may impact their ability to work (e.g., per [21]. Their non-clinical assessment of their work ability may be an early indicator of possible deteriorating health conditions [22]. Other work drivers, such as accidents at work, can be accounted for by specifically considering whether people had time off on workers’ compensation in the year prior to the target window.

Further, although health status appears to be more important in determining health service utilization than health insurance [23], in some countries, health insurance coverage may either directly impact health care utilization through availability or as a self-selection effect [24]. Similarly, along with whether the potential patient has private health insurance (PHI), some people may have a form of health care card (mostly regarding being military veterans for this sample’s age range) that may reflect effects similar to that of PHI, such as a self-selection effect of being more health aware and/or the availability of hospital access (whether paid for or service-related). The substantial and growing waiting times for hospitalization, whether for emergency or elective procedures, may also reflect socioeconomic inequities [25], and those with insured access may be able to avail themselves of elective procedures before their health condition deteriorates to the point of needing emergency care [26]. Finally, among those living alone, health indicators may be affected by whether they are divorced, never married or widowed [3] and possibly by sex and age (as often controlled for in health trajectory analyses of mortality (e.g., per [16])).

This study will longitudinally investigate a cumulative health model of hospitalization for those who live alone. A baseline of health status will reflect whether the individual has been clinically advised of their having a serious illness four years prior to the potential hospitalization window. Other health information that the individual would know is then assessed, especially in terms of their ability to work and their physical functioning, among other health issues, while also considering indicators of various sub-groups in terms of PHI or similar and accounting for age, sex and the nature of their living alone status (divorced or not).

## 2. Materials and Methods

The Household, Income, and Labor Dynamics in Australia (HILDA) dataset, with Melbourne University ethics approval number 1647030, was the source of the sample’s data. After an initial sampling intended to be representative of all Australian households, the HILDA survey team uses a set of processes and rules to endeavor to maintain representativeness over time (the processes are detailed in [27]).

A layering approach to the consideration of the development of health conditions was used. The serious illness categorizations were coded at Time 1, four years prior to the hospitalization window. The more recent indications of deteriorating conditions likely to be known by the potential patient or their primary care clinician were coded at Time 2, one year prior to the target window. The contextual, non-health drivers were assessed at Time 3, in parallel with the possible hospitalization window. HILDA works on a rotating modules system where detailed questions about certain topics are only asked every four years. Consequently, detailed health measures, including those regarding having been clinically advised of serious illnesses and of hospitalization, are asked only every four years. Using the data released in early 2022, the most recent year (Time 3) with the detailed health questions was 2017. The variables that appeared to have strongly non-linear relationships were coded to represent that non-linearity.

At Time 1, respondents indicated whether they had been told by a doctor or a nurse that they had one or more conditions from a list of serious illnesses (a different set of conditions, although overlapping with the chronic list used previously) or had none of those conditions. Those indicating that they had type 2 diabetes, cancer, high blood pressure/hypertension, arthritis/osteoporosis, depression or anxiety were categorized as having a low to moderate relative risk of hospitalization. Those respondents indicating that they had heart disease, asthma, other mental illness, any other serious circulatory condition (e.g., stroke, hardening of the arteries), type 1 diabetes, and/or chronic bronchitis or emphysema were categorized as having a high relative risk of hospitalization. Respondents were also coded in terms of comorbidity across this list of 11 conditions if they had more than one of those conditions.

However, because both the severity and comorbidity indicators had a common category (those with none of those conditions), the indicators of severity and comorbidity were combined so as to also reflect the interaction between high severity and comorbidity. The resulting combined variable (Hospitalization Severity with Comorbidity) was coded from no severe illness (0) to (1) having low hospitalization severity with comorbidity to (2) a low to high relative hospitalization rate with no comorbidity and (3) a high relative hospitalization rate with comorbidity.

The respondents also indicated whether they had (1 yes/2 no) a long-term health condition that restricts their everyday activities from among a list of chronic conditions and impairments that could impact their ability to work. Examples from the set of conditions included those that restrict physical activity, such as back problems, mental illnesses that require assistance or supervision and other long-term conditions, such as arthritis or Alzheimer’s (long-term health condition, Time 1).

Three years later, at Time 2, the participants were again asked to indicate whether they had a long-term health impairment that could impact their ability to work, and this time, they were also asked whether the impairment or condition impacted the amount or type of work they could do. Those respondents indicating that they had a condition that limited the amount or type of work they could do then indicated, from zero—not at all, to ten—unable to work, the degree to which their condition limited the work they could do (work ability, Time 2). Respondents were grouped such that those with no long-term health condition or a condition that did not limit their work or scored only one out of ten were coded as (0), no work ability impact. The respondents answering two to ten for the degree of work impact were coded as (1), some impact.

A suite of global health measures were used at Time 2, a year before the hospitalization target window. Respondents were scored for the physical functioning, general health and mental health sub-scales of the SF36 on the 0–100 index [17]. The three indices had non-linear relationships with the outcome variables and consequently were coded to try to reflect those non-linear relationships. The physical functioning scale was coded as less than 85 versus greater than or equal to 85. The general health scale was coded as less than 60 versus greater than or equal to 60, and the mental health scale was coded as less than or equal to 40 relative to greater than 40. Further, in order to be able to separate out those suffering from a work-related injury, respondents were asked at Time 2 to indicate whether they had spent any time on workers’ compensation in the last 12 months (1, yes; 2, no).

Demographic variables were assessed as at Time 3 with participants indicating their sex (1 male, 2 female) and age at their last birthday as of 30 June 2017. The participants’ divorced or widowed/single status was derived from a question asking about their current marital status. There were no responses of married or living with someone in a relationship for this sample living alone. Note that the legal system in Australia is such that those getting divorced can be required to stay separated for one year before the divorce proceedings can be finalized, and separated but not divorced status may continue for some time if one party is unable to pay for the paperwork to be put through when the other party is not willing to finalize the paperwork. Further, couples that dissolve a de facto relationship are sometimes called separated rather than divorced. Thus separated and divorced were combined. The responses of widowed and never married and not living with someone in a relationship were also combined.

At Time 3, the respondents also indicated whether they had private health insurance (PHI) and what form of health insurance they had. Note that all Australian residents are covered by the Australian form of Medicare and that any private health insurance would be on top of Medicare coverage. The question asked, apart from Medicare, are you currently covered by private health insurance? If the respondent indicated yes, they were asked what type of health insurance they had. The responses were coded (0) hospital cover only or both hospital and extra coverage, or (1) no private health insurance or extra coverage only. A final indication of special groups that were controlled for were those groups with access to Department of Veterans’ Affairs Orange, White or Gold Treatment Entitlement Cards, Health Care Cards, Pensioner Concession Cards, and Commonwealth Seniors Health Cards relative to those who had none of these cards. Finally, at Time 3, respondents were asked: during the last 12 months, have you been admitted as a patient to a hospital for an overnight stay (1, yes; 2, no).

## 3. Results

The starting sample of 1086 respondents matched three times over 4 years had 67 cases with one or more missing values (mostly on the SF36 variables), leaving 1019 cases for the main analyses. The missing data had cases that were assessed to be missing completely at random (MCAR) with Little’s MCAR test (*p* = 0.475). Further analyses at the end of this section also assessed the impact of the missing values. The summary results describing the distribution of the variables across the hospitalization categories as at Time 3 (n = 125 hospitalized in last 12 months, n = 894 not hospitalized in last 12 months) are as detailed in Table 1.

Logistic regression was conducted on the data using SPSS version 28 following [28]. Box–Tidwell checks on age indicated that curvilinear transforms of age would not have enhanced the analyses. The logistic regression model had −2LL = 642.893, a significant improvement over the default model’s 719.041, with χ^2^ (14) = 106.148, *p* < 0.001 and a Nagelkerke R-squared of 0.188. The odds ratios and logit parameter estimates are shown in Table 2, where the comparison state is not having overnight hospitalization at Time 3.

**Figure 1:**
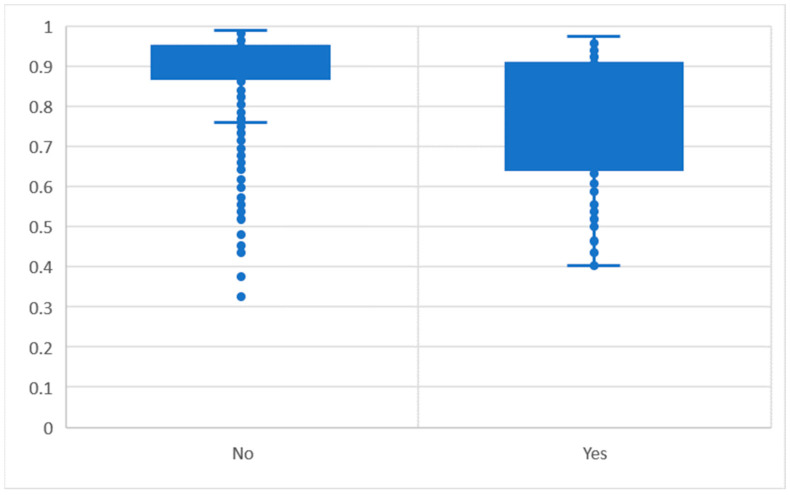
The predicted probability by actual overnight hospitalization status.

The significant variables were having a long-term health condition (Time 1), hospitalization severity and comorbidity (Time 1), work ability (Time 2), physical functioning (Time 2), single status (Time 3) and having one or more health care cards. Post hoc power analyses using G*Power 3.1 [29] with effective odds ratios over 1.6 and the sample details above calculated the power to be 1.0. Having hospital coverage in their private health insurance also had a tendency to predict overnight hospitalization. The chart of the logistic regression results is summarized in Figure 1.

Repeating the above analyses after replacing cells with missing data across 40 sets of multiply-imputed data with fully conditional specification found similar results to those in Table 2. The same variables were significant, except that the PHI variable had moved from significant to *p* < 0.10, and the combined Severity and Comorbidity variable from Time 1 became significant at *p* < 0.05.

## 4. Discussion

This study extends the limited, conflicting evidence as to whether living alone impacts the utilization of health services [6] by confirming that key characteristics of those living alone do predict the use of health services, particularly in this case, overnight hospitalization of the under-studied cohort of those aged 30 to 64 years of age who are living alone. The key drivers of overnight hospitalization for those who live alone were high hospitalization severity and comorbidity at Time 1, having a long-term health condition at Time 1, some impact of work ability at Time 2, poor physical functioning at Time 2, being separated/divorced (at Time 3) and having one or more health care cards. Having hospital coverage in their private health insurance also had a tendency to predict overnight hospitalization. These findings suggest that a cumulative health deterioration model is useful for predicting overnight hospitalization and that there are a variety of special groups that may need to be separately considered or parsed out of analyses due to the potentially differential access to hospitalization (e.g., due to private health insurance for hospitalization or service-related access to hospitalization).

For this cumulative approach to health modelling, indicators of both the more temporally distant clinically-advised serious illnesses and the temporally proximal functional health measure and functionality in terms of work ability were significant. In terms of the more temporally distant health indicators, having a long-term condition, even one that could be managed with primary care (per [11]), along with severe, usually more acute, illnesses that have often been treated in a hospital, provide a baseline as to the likely need for overnight hospitalization up to four years later. These classifications of the severity of the risk of hospitalization for those living alone may also be indications of dysfunctions in the healthcare system [14], such as the lack of efficacy of certain treatment programs, the hesitancy of people with certain illnesses to have frequent contact with primary care and the potential obstacles to, or delays with, accessing primary care.

Over time, that baseline risk of hospitalization can be added to in terms of more proximal health indices such as self-rated work ability and measures of physical functioning. In a similar manner to studies of the elderly where physical functioning was predictive of hospitalization [19,20], physical functioning was predictive of hospitalization for these second-age adults living alone. The predictive utility of an index of physical functioning, as well as simple self-assessments of whether their health could impact their ability to work, suggests that the salience to a lay individual of their physical functioning in terms of their ability to work and to their general practitioner of their global physical functioning could be acted upon. The salience of functionality to the individual, assessed relative to a standard they are familiar with, their ability to work, is a finding that highlights the importance of such assessments of work ability, supporting and extending [21,22].

Accounting for or separating out special sub-groups for specific health trajectories, in this case to overnight hospitalization, may make it easier to determine the key drivers of hospitalization for the remainder of the sample of interest. For example, alternate drivers of hospitalization, such as accidents at work, can be accounted for by specifically considering whether people had time off on workers’ compensation in the year prior to the target window. Similarly, PHI appears to directly impact overnight hospitalization through the availability of insured funding for hospital care [25] or as a self-selection effect of those expecting hospitalization (confirming [24]), as does the access to special health care cards such as those of military veterans. These are examples of specific-issue groups that should be accounted for when assessing health trajectories, especially for forms of hospitalization. Further, the findings suggest that second-age people living alone used PHI and health care cards to access hospitalization and were not passively waiting for someone to take them to health services.

More generally, by focusing on studying those living alone as a proxy for social isolation [11], the pattern of the findings above can suggest which of the two main mechanisms for the impact of living alone on overnight hospitalization appears to be stronger. Either the social isolation is clinically leading to poor health and increased health needs and/or, more instrumentally, those living alone may require increased support during episodes of ill health (per [6]). The strength of the health results above suggests that the impact of social isolation on overnight hospitalization is more instrumental, where those living alone are less able to manage an episode of ill health and, with a lack of options for others to take them to primary or other care when they are ill, those living alone with health conditions go directly to the hospital, possibly inappropriately. Further, those living alone, facing the lack of flexibility and options for going through the stages of the health system, may also delay accessing health care until the illness is quite bad and then end up overnight in the hospital.

An addendum to this suggestion of the instrumental mechanisms of the impact of social isolation or support is reflected in the finding that being separated/divorced had higher rates of overnight hospitalization than those living alone who were single or widowed (partially clarifying and confirming [3]). Those living alone who are separated/divorced are likely to have had their social support systems disrupted and may, perhaps only temporarily, but perhaps not, have lower levels of social support or higher levels of social isolation than those who have never married.

### Limitations

The main limitation of this study is that the data analyzed were mostly either self-reported or from the interviewer (household type—whether living alone). Future research may wish to incorporate objective indices with such self-report data to extend the results found above. The results above would not be broadly generalizable, because they focus on those living alone, but there is a dire need for more studies on those living alone, and such consideration of groupings in the population may also help to facilitate more customized approaches to care.

The nature of one of the key variables, serious illnesses and comorbidity, was based on the respondents’ recollection of having been clinically advised of those illnesses. For this paper, the emphasis was more on issues the potential patient may know that may also act as prompts for their general practitioners. Future research may want to incorporate more objective data, such as data from registries.

Recall bias is unlikely given the long time between measurements and because each of the measures was contemporaneous to that time (that is, the analyses may have occurred after the data were collected, but the measures of the variables were prospective from the point of view of the respondents). Further, the separation in time between the drivers and target window avoided biasing the results, as has occurred in other studies of hospitalization [3].

Another potentially key limitation is that the pattern of results above was specific to the Australian context, a context with relatively universal healthcare and a comprehensive welfare system. Thus, future studies may want to investigate drivers of overnight hospitalization for those who live alone in other contexts.

## 5. Conclusions

With large and growing numbers of citizens living alone in developed countries such as Australia and the substantial costs associated with hospital care, there needs to be a better understanding of the health service utilization of those living alone [6], particularly in efforts to reduce their higher mortality risk [16]. The growing rates of living alone and hospitalization suggest that the increasing presence of technology has not necessarily helped reduce social isolation. The notable findings of this study confirm the viability and need to study those living alone in post-education adulthood as a distinct group.

The strength of the various health results above suggests that the impact of living alone on overnight hospitalization is more instrumental, where those living alone are less able to manage an episode of ill health, and, with a lack of options for others to take them to primary or other care when they are ill, those living alone with health conditions go directly to a hospital. The instrumental nature of hospitalization for those living alone may also be impacted by disruptions to social systems due to separating or getting divorced.

Importantly, the accumulation of health issues was predictive up to four years before the hospitalization window. The baseline risk of hospitalization due to serious illnesses is modified as symptoms and relatively salient changes in functionality accumulate. Further, accounting for or separating out special sub-groups for health trajectories, such as overnight hospitalization, may make it possible to determine the key drivers of hospitalization for the remainder of any given sample of interest.

The conditions under which overnight hospitalization occurs for these working-age adults living alone and the mechanisms by which living alone impacts hospitalization suggest that hospital settings may not be the most cost-effective or clinically optimal setting to meet some of their health needs. The findings of this study could inform appropriate interventions, especially with some of the drivers of hospitalization potentially being preventable with primary care, especially in terms of making primary care available and accessible. Working-age adults living alone and their general practitioners could use changes in functionality as an early warning sign and act to intervene.

## Figures and Tables

**Table 1:** The variables in the analyses by hospitalization status at time 3.

	Hospitalized Overnight in Last 12 Months (Time 3), n = 1019	
Categorical Variables	Yes (n)	No (n)
Hospitalization severity with comorbidity (Time 1)		
- No severe illness	39	492
- Low hospitalization severity and comorbidity	11	67
- Low and high severity with no comorbidity	38	236
- High hospitalization severity and comorbidity	37	99
Long-term health condition (Time 1)		
- Yes	63	282
- No	62	612
Work ability (Time 2)		
- No impact	55	712
- Some impact	70	182
Workers’ compensation last 12 months (Time 2)		
- Yes	9	19
- No	116	875
Physical functioning (Time 2)		
- LT85	80	260
- GTE85	45	634
General health (Time 2)		
- LT60	76	320
- GTE60	49	574
Mental health (Time 2)		
- LTE40	26	77
- GT40	99	817
Sex (Time 3)		
- Male	62	488
- Female	63	406
Partnered status (Time 3)		
- Separated/divorced	66	373
- Widowed and never married	59	521
PHI (Time 3)		
- Hospital only or hospital with extras	49	404
- No PHI or PHI for extras only	78	490
Health care cards (Time 3)		
- Yes, have 1 or more cards	60	199
- No, have none of those cards	65	695
Continuous Variables	Mean (Standard Deviation)	
Age (Time 3)	51.34 (9.889)	49.12 (10.397)

**Table 2:** The parameters of the logistic regression results for having overnight hospitalization at Time 3.

	Hospitalized Overnight in Last 12 Months (Time 3), n = 1019		
Categorical Variables	B (S.E.)	Odds Ratio	95% C.I.
Hospitalization Severity with Comorbidity (Time 1)			
- No severe illness	−0.877 (0.340)	0.416 *	0.214–0.810
- Low hospitalization severity and comorbidity	−0.795 (0.408)	0.452 ^†^	0.203–1.005
- Low and high severity with no comorbidity	−0.476 (0.304)	0.621	0.343–1.127
- High hospitalization severity and comorbidity			
Long-term health condition (Time 1)			
- Yes	−0.814 (0.305)	0.443 **	0.244–0.805
- No			
Work ability (Time 2)			
- No impact	−0.975 (0.305)	0.377 **	0.207–0.687
- Some impact			
Workers’ compensation last 12 months (Time 2)			
- Yes	1.127 (0.466)	3.087 *	1.238–7.696
- No			
Physical functioning (Time 2)			
- LT85	0.969 (0.276)	2.634 **	1.533–4.527
- GTE85			
General health (Time 2)			
- LT60	0.075 (0.258)	1.078	0.651–1.787
- GTE60			
Mental health (Time 2)			
- LTE40	0.222 (0.300)	1.248	0.693–2.248
- GT40			
Sex (Time 3)			
- Male	0.094 (0.214)	1.099	0.723–1.670
- Female			
Single status (Time 3)			
- Separated/divorced	0.481 (0.223)	1.618 *	1.044–2.506
- Widowed and never married			
PHI (Time 3)			
- Hospital only or hospital with extras	0.425 (0.244)	1.530 ^†^	0.948–2.467
- No PHI or PHI for extras only			
Health care cards (Time 3)			
- Yes, have 1 or more cards	0.635 (0.288)	1.886 *	1.073–3.316
- No, have none of those cards			
Age at Time 3 (Z)	−0.128 (0.130)	0.880	0.682–1.135
Constant	−1.663 (0.503) **		

Note: ^†^ < 0.10, * < 0.05, ** < 0.01. S.E. = standard error, C.I. = confidence interval, GTE = greater than or equal to, LTE = less than or equal to, GT = greater than, and LT = less than. The reference categories (blank lines) were set to 0.

## Data Availability

The HILDA data are available and managed by the Melbourne Institute of Applied Economic and Social Research at The University of Melbourne (https://melbourneinstitute.unimelb.edu.au/hilda accessed on 29 September 2022). The version used above was version 21.
